# Ferroptosis and prostate cancer: A translational path from molecular mechanisms to precision therapy

**DOI:** 10.1016/j.gendis.2025.101967

**Published:** 2025-12-08

**Authors:** Yixiang Huang, Yuanxin Ma, Jiachen He, Tanjing Song

**Affiliations:** a Tongji Hospital, Tongji Medical College, Huazhong University of Science and Technology, Wuhan, Hubei 430030, China; b First Clinical College, Tongji Medical College, Huazhong University of Science and Technology, Wuhan, Hubei 430030, China; c Department of Biochemistry and Molecular Biology, School of Basic Medicine, Tongji Medical College, Huazhong University of Science and Technology, Wuhan, Hubei 430030, China; d Cell Architecture Research Institute, Huazhong University of Science and Technology, Wuhan, Hubei 430030, China

**Keywords:** Combination therapy, Ferroptosis, Ferroptosis-inducing compounds, Lipid peroxidation, Prostate cancer

## Abstract

Castration-resistant prostate cancer represents a critical clinical challenge due to its propensity for resistance to conventional therapies and limited treatment efficacy. Ferroptosis is an iron-dependent form of programmed cell death driven by lipid peroxidation. It holds therapeutic potential and can be induced by glutathione peroxidase 4 (GPX4) inhibition, glutathione depletion, or iron overload using compounds such as RSL3 and Erastin. These approaches show promise in overcoming drug resistance and enabling synergistic effects with anti-androgen therapy, chemotherapy, and immunotherapy. This review systematically summarizes the core regulatory networks of ferroptosis in prostate cancer (such as the PI3K–AKT–mTOR, Hippo/YAP, PGE2, and their downstream pathways), summarizes combination treatment strategies and clinical trial progress, proposes a three-pronged translational framework of “ferroptosis regulatory network–biomarkers–precision therapy”, and discusses the challenges it faces in terms of drug resistance, targeting accuracy, and clinical translation. These insights aim to accelerate biomarker discovery, optimization of multimodal combination regimens, and the translation of ferroptosis from fundamental research into transformative therapeutic interventions.

## Introduction

Prostate cancer, a significant malignant neoplasm affecting men, is a leading contributor to increased mortality rates among males worldwide.^1^ According to data on the global burden of cancer in 2020 published by the International Agency for Research on Cancer (IARC) of the World Health Organization, prostate cancer ranks second in incidence among male cancers. On a global scale, the incidence and mortality of prostate cancer are projected to continue to increase in the coming decades.^2^

Currently, the primary therapeutic modalities for prostate cancer encompass surgical resection, androgen deprivation therapy, radiotherapy, endocrine therapy, and chemotherapy.^3^ However, for advanced metastatic prostate cancer, particularly castration-resistant prostate cancer (CRPC), these conventional treatment methods frequently prove ineffective. Despite undergoing traditional androgen deprivation therapy, the disease continues to progress rapidly, developing multiple drug resistances. Therapeutic options for patients diagnosed with CRPC are severely limited, and their 5-year survival rate remains low. Consequently, a pressing need exists within the prostate cancer research community to understand in-depth the pathogenesis of prostate cancer and to identify novel and effective therapeutic targets and strategies.

Ferroptosis, a recently discovered mode of programmed cell death, is distinguished by the accumulation of lipid peroxidation products on the cell membrane, catalyzed by iron ions. Membrane fluidity is crucial for cells, and it is associated with the lipids within the cell membrane, which contain a high proportion of unsaturated fatty acids; however, Lipid peroxidation on the membrane makes the cell membrane fragile, ultimately resulting in membrane rupture and cell death.^4^ A growing body of research has demonstrated the sensitivity of prostate cancer cells with metastatic potential to ferroptosis.^5^ The enhancement of tumor cells' response to therapy through the modulation of ferroptosis sensitivity offers novel therapeutic strategies for tumor treatment.

## Regulatory mechanisms of ferroptosis in prostate cancer

### Core metabolic pathways

The central mechanism of ferroptosis, an iron-dependent form of programmed cell death, involves a dynamic balance between iron overload-driven lipid peroxidation and the multiple antioxidant defense systems.^6^^,^^7^

The initiating factor for ferroptosis is impaired iron metabolism. Extracellular iron enters the cell by endocytosis via transferrin receptor 1 (TFR1) and is catalyzed by six-transmembrane epithelial antigen of prostate 3 (STEAP3) or Haber–Weiss reaction, where Fe^3+^ is reduced to Fe^2+^, which is subsequently transported by divalent metal transporter 1 (DMT1) to the labile iron pool in the cytoplasm.^4^^,^^8^ Under iron overload conditions, Fe^2+^ reacts with hydrogen peroxide (H_2_O_2_) via the Fenton reaction to generate highly reactive hydroxyl radicals (·OH), which initiate the lipid peroxidation chain reaction of polyunsaturated fatty acids (PUFAs) in the plasma membrane phospholipids, leading to loss of cell membrane integrity and organelle dysfunction, and ultimately triggering ferroptosis.^6^^,^^9^ Cysteine desulfurase nitrogen fixation 1 (NFS1) is a key rate-limiting enzyme for the biosynthesis of iron-sulfur clusters in mitochondria, and its insufficient activity triggers mitochondrial iron overload, causing reactive oxygen species (ROS) accumulation and triggering ferroptosis.^10^

In the event of the existence of such mechanisms that promote ferroptosis, it is logical to infer the existence of reductive mechanisms that counteract ferroptosis. In contrast to pro-death mechanisms driven by iron overload, the Xc^−^ system represents a key antioxidant defense pathway. This transporter, a heterodimer composed of solute carrier family 7 member 11 (SLC7A11) and solute carrier family 3 member 2 (SLC3A2), facilitates the import of extracellular cystine into the intracellular space, where it is reduced to cysteine.^11^ Cysteine serves as a precursor for the synthesis of glutathione (GSH).^4^^,^^6^^,^^7^ Ferroptosis suppressor protein 1 (FSP1), in contrast, is a GSH-independent inhibitor of ferroptosis that has been discovered in recent years. The process entails the reduction of ubiquinone (CoQ_10_) to ubiquinol (CoQ_10_H_2_) through an NADH/NADPH-driven reduction reaction. The latter functions as a lipid radical scavenger, thereby inhibiting the phospholipid peroxidation chain reaction and, consequently, preventing ferroptosis.^12^^,^^13^ As indicated by the numerical values, the function of FSP1 is contingent upon its N-terminal myristoylation modification. This modification facilitates its localization to the plasma membrane and underlies its antioxidant function within the lipid environment.^12^ Dihydroorotate dehydrogenase (DHODH) and FSP1 both reduce lipid peroxidation levels by reducing ubiquinone, thereby antagonizing ferroptosis.^14^ Additionally, GTP cyclohydrolase 1 (GCH1) serves as a key enzyme in tetrahydrobipterin (BH4) synthesis, playing a crucial role in this process. BH4, acting as a reducing equivalent, can reverse lipid peroxidation and thereby counteract ferroptosis.^14^ Vitamin K, as a reducing equivalent that humans can ingest, also exhibits certain anti-lipid oxidation functions.

**Figure 1:**
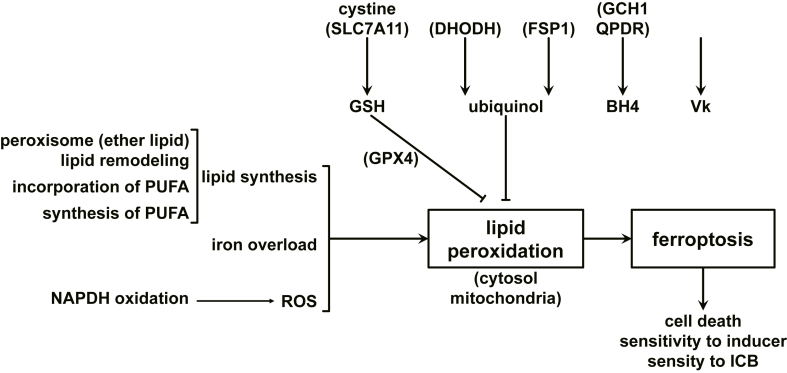
Core pathways of ferroptosis in prostate cancer.

The key to ferroptosis is iron accumulation and the subsequent lipid peroxidation that it causes, which promotes ferroptosis. However, multiple pathways can also counteract this process, demonstrating the body’s capacity for recovery. It has been demonstrated that all ferroptosis pathways converge on this mechanism. Nevertheless, the regulatory mechanisms in question are characterized by a high degree of complexity. Inducing ferroptosis also triggers various compensatory mechanisms, which consequently lead to therapeutic challenges. The core concepts pertain to iron accumulation and overload, lipid peroxidation, and ROS accumulation, in addition to the central redox-antioxidant mechanism, as illustrated in Figure 1.

### Key regulatory factors

Research on the molecular mechanisms of the ferroptosis regulatory network in prostate cancer has made remarkable progress in recent years, and its core pathway involves iron metabolism disorders, lipid peroxidation imbalance, and synergistic regulation of key signaling pathways.

At the level of iron homeostasis regulation, TFR1 increases the intracellular iron pool by mediating cytosolic iron uptake, whereas membrane iron transport proteins maintain the dynamic balance of iron metabolism through iron efflux.^15^, ^16^, ^17^ In addition, nuclear receptor coactivator 4 (NCOA4) further amplifies the ferroptosis-associated oxidative stress effect by mediating the autophagic degradation of ferritin heavy chain 1 (FTH1), which prompts the release of stored iron into unstable iron pools.^6^^,^^18^

The regulatory network of aberrant lipid metabolism is a complex and antagonistic system that acts as a direct driver of ferroptosis. PUFAs promote ferroptosis by increasing the oxidative sensitivity of membrane phospholipid bilayers.^14^^,^^15^^,^^19^ In particular, acyl coenzyme A synthase long-chain amide member 4 (ACSL4) promotes the integration of PUFA into membrane phospholipids and increases oxidative susceptibility by catalyzing the formation of PUFA-CoA.^14^^,^^15^^,^^20^ Loss of function of diacyl coenzyme A reductase 1 (DECR1), the rate-limiting enzyme for PUFA β-oxidation, leads to abnormal accumulation of PUFAs and triggers ferroptosis.^19^^,^^21^ Some pathways protect PUFAs from oxidation: cells maintain PUFA structural stability from oxidation through the GCH1–BH4 axis.^22^ In contrast, monounsaturated fatty acids, on the other hand, play a role in protecting cell membranes by competitively integrating into cell membrane phospholipids.^15^ Stearoyl coenzyme A desaturase 1 (SCD1) antagonizes the integration of PUFAs into the cell membrane by converting saturated fatty acids to monounsaturated fatty acids.^23^^,^^24^

In addition, there are also pathways that inhibit ferroptosis by antagonizing lipid oxidation. Phospholipase A2G4A (PLA2G4A) inhibits ferroptosis through the release of arachidonic acid, whose metabolite prostaglandin E2 (PGE2) can exert a significant anti-ferroptosis effect.^15^ BH4 also works synergistically with coenzyme Q10 (CoQ10) and vitamin E to protect lipid membranes from autoxidation.^22^^,^^25^ In addition, the abnormal accumulation of squalene (squalene) in some cancer cells can act as an intramembrane natural antioxidant barrier in the absence of the key enzyme for cholesterol synthesis, squalene epoxidase (SQLE), effectively blocking the triggering of ferroptosis.^26^ These glutathione peroxidase 4 (GPX4) non-dependent protective mechanisms provide new metabolic entry points for the precise regulation of cellular ferroptosis pathways and the development of anti-tumor targeting strategies.

The interactive regulation of key signaling pathways provides a fine molecular switch for ferroptosis. Loss of phosphatase and tensin homologue (PTEN) is prevalent in prostate cancer. The loss of this gene activates the phosphoinositide 3-kinase (PI3K)–protein kinase B (AKT)–mammalian target of rapamycin (mTOR) pathway, which promotes the maturation of sterol regulatory element-binding protein 1 (SREBP1). Subsequently, SREBP1 activates the transcription of SCD1, ATP-citrate lyase (ACLY), acetyl-CoA carboxylase alpha (ACACA), and fatty acid synthase (FASN), thereby establishing a robust anti-apoptotic barrier. A key effector of this pathway is SCD1, which catalyzes the conversion of saturated fatty acids into monounsaturated fatty acids, thereby preventing ferroptosis.^23^ In addition to the PI3K–AKT–mTOR pathway, the Hippo/YAP/TAZ pathway plays a critical and complex role, exhibiting bidirectional regulatory properties: Under physiological conditions, the Hippo signaling pathway is activated, and YAP/TAZ are phosphorylated and located in the cytoplasm, where they are degraded via the ubiquitin–proteasome pathway, thereby maintaining tissue homeostasis.^7^^,^^20^^,^^27^^,^^28^ When Hippo is inactivated, YAP/TAZ is dephosphorylated and translocated to the cell nucleus, activating signals involved in cell proliferation regulation, and can inhibit oxidative stress through the activating transcription factor 4 (ATF4)–SLC7A11–GPX4 axis.^29^ Some key molecules can also regulate the Hippo/YAP pathway. For example, the deubiquitinating enzyme CYLD enhances the pro-ferroptotic effect of YAP protein by stabilizing it, while the androgen receptor (AR) reshapes lipid metabolism homeostasis by up-regulating acyl-CoA synthetase long chain family member 3 (ACSL3) and inhibiting ACSL4. However, inhibiting AR itself will also cause adaptive responses such as phosphoglycerate dehydrogenase (PHGDH) up-regulation, revealing the complexity and timing importance of targeting AR in combination with ferroptosis.^20^ Research has found that in prostate cancer mouse models with PTEN and Smad4 defects, YAP directly induces C-X-C motif chemokine ligand 5 (CXCL5) expression to attract myeloid-derived suppressor cells expressing CXCL2, thereby suppressing tumor immunity. This indicates that the PI3K–AKT–mTOR pathway and Hippo/YAP/TAZ are not isolated; they share common upstream factors that participate in the co-regulation of prostate cancer. P53, as a classic tumor suppressor gene, also exhibits dual regulatory properties. On one hand, it inhibits SLC7A11 to restrict cysteine uptake, thereby promoting ROS accumulation. On the other hand, it modulates protease function to attenuate the ferroptosis effects induced by Xc^−^ inhibitors such as Erastin. P53 deficiency also reduces cellular sensitivity to Erastin, making the selection of p53 status critical. Additionally, selecting RSL3 to induce ferroptosis by inhibiting GPX4 may be more effective.^6^^,^^30^^,^^31^ It is worth noting that AR may not only directly affect lipid metabolism but may also influence the Hippo/YAP pathway or p53 function through mechanisms that have not yet been fully elucidated, further increasing the complexity and specificity of the ferroptosis regulatory network in prostate cancer.

**Figure 2:**
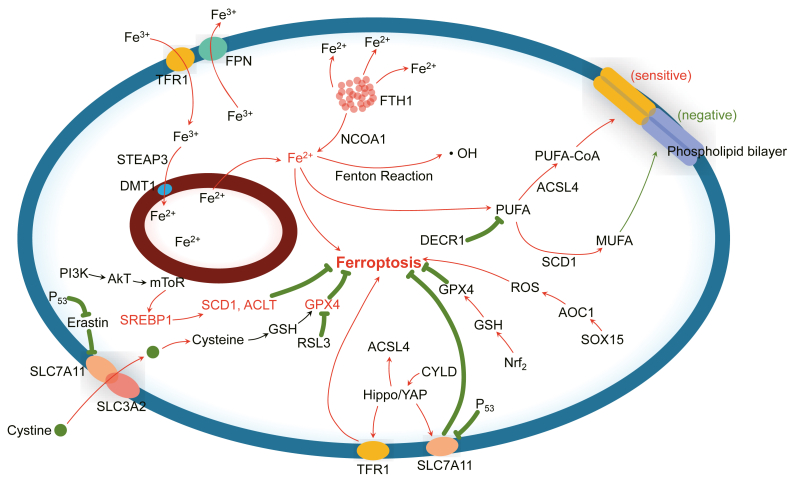
Schematic representation of ferroptosis pathways and regulatory mechanisms.

Recent studies have also revealed a key role in the oxidative stress defense system, with nuclear factor erythroid 2-related factor 2 (Nrf2) significantly enhancing cellular antioxidant capacity through activation of the GSH–GPX4 axis.^6^^,^^32^^,^^33^ Meanwhile, SRY-box 15 (SOX15) promotes ROS generation from spermine metabolism through transcriptional activation of amine oxidase copper-containing 1 (AOC1), forming a unique pro-ferroptosis loop.^34^ It has also been shown that SOX11 is one of the regulators of ferroptosis.^35^ Figure 2 systematically depicts the regulatory network of ferroptosis in prostate cancer by integrating iron metabolism, lipid metabolism, and key signaling pathways. This schematic diagram clearly reveals the intricate interactions among these pathways, providing a crucial theoretical framework for understanding ferroptosis’s dual regulatory role in prostate cancer and its potential therapeutic value.

Non-coding RNAs and gene regulation also serve as important mediators of epistatic regulation. miR-9 synergistically promotes ferroptosis by decreasing GSH levels, miR-1252-5p synergistically promotes ferroptosis by repressing FTH1 expression, and LINC00336 exerts a protective effect by repressing ferroptosis-related genes.^6^^,^^16^ These findings provide a multidimensional perspective for in-depth analysis of the ferroptosis regulatory network in prostate cancer and a theoretical basis for the development of targeted therapeutic strategies. SRC, AIF family member 2 (AIFM2), SLC2A6, heme oxygenase 1 (HMOX1), neuroglobin (NGB), metallothionein 1G (MT1G), dopamine receptor D4 (DRD4), centrosome and spindle pole associated protein 1 (CSPP1), and aldo-keto reductase family 1 member C2 (AKR1C2) genes and proteins are associated with the prostate cancer ferroptosis pathway by univariate and multivariate Cox regression analysis.^36^^,^^37^

Some enzymes in the synthetic pathway were also found to be highly expressed in prostate cancer cells, such as PHGDH. PHGDH is a key enzyme in the serine synthesis pathway, and the expression of PHGDH and its downstream genes (*e.g.*, PSAT1, PSPH, and DHFRL1) was significantly higher in enzalutamide-resistant CRPC cells than in sensitive cells.^38^ These enzymes can all serve as important markers for the future development of ferroptosis therapy detection systems. There are numerous regulatory mechanisms for ferroptosis.

The key regulatory mechanisms and important omics data mentioned above represent pivotal points in ferroptosis. Pathways such as TFR1, along with key omics data such as SLC7A11, have been included in Table 1 for ease of reference and comprehension.

**Table 1:** Important omics data in ferroptosis.

Omics type	Key molecule	Regulation	Functional mechanism	Experimental model
Genomics	GPX4 deletion	↓	Loss of glutathione peroxidase activity → lipid ROS accumulation	TRAMP mouse model
	ACSL4 amplification	↑	Promotes poly-unsaturated fatty acid synthesis → increased lipid peroxidation sensitivity	Patient tumor tissues
Transcriptome	SLC7A11 inhibition	↓	Impaired cystine uptake → decreased GSH synthesis	LNCaP/PC-3 cell lines
	Nrf2 pathway activation	↑	Up-regulates HO-1, FTH1 → resistance to oxidative damage	Castration-resistant PDX model
Proteomics	p53 phosphorylation	↑	Inhibits SLC7A11 expression; promotes SAT1–ALOX15 pathway	22Rv1 cell line
	HSPB1 overexpression	↑	Suppresses iron transport → blocks Fenton reaction	Clinical sample mass spectrometry
Metabolomics	Polyunsaturated fatty acids ↑	↑	Accumulation of lipid peroxidation substrates → Elevated MDA/4-HNE levels	Patient serum metabolomics
	Glutathione (GSH) ↓	↓	Collapse of antioxidant system → loss of ROS scavenging capacity	Tissue metabolic fingerprinting

### Regulation of ferroptosis by the tumor microenvironment

There is a complex bidirectional regulatory relationship between the immune microenvironment and ferroptosis in prostate cancer. Studies have shown that CD8^+^ T cells induce ferroptosis in tumor cells by activating the Janus kinase (JAK)–signal transducer and activator of transcription (STAT1) pathway through the secretion of interferon-γ (IFN-γ) and inhibiting expression of the cystine transporter SLC7A11, leading to GSH depletion and accumulation of lipid peroxidation. This process can be further enhanced by anti-programmed death-1 (PD-1) therapy, creating a positive immune loop.^7^^,^^39^ Notably, the M2-type subpopulation of tumor-associated macrophages inhibits ferroptosis and maintains an immunosuppressive microenvironment by activating the liver X receptor α (LXRα)/SCD1 pathway through the secretion of taurine, a process that can be reversed by targeting the taurine transporter protein TauT or by blocking LXRα signaling.^24^

The immunogenic character of ferroptosis unveils novel therapeutic avenues. Ferroptosis inducers (*e.g.*, RSL3) activate dendritic cells and M1-type macrophages by releasing damage-associated molecular patterns (DAMPs) to promote antigen presentation and T-cell infiltration. Radiation therapy-mediated particles loaded with ferroptosis inducers and apoptosis-inducing peptides synergistically activate the STING pathway and remodel the immune microenvironment to enhance the anti-tumor immune response.^38^^,^^40^

Recent studies have shown that prostaglandin E2 (PGE2) prevents the value-added of tumor-infiltrating lymphocytes by binding to the receptor EP2/EP4 to activate calcium ions, degrading the interleukin-2 (IL-2) receptor γ-chain (IL-2Rγc), and blocking the downstream signaling of IL-2, and that tumor-infiltrating lymphocytes are unable to exert anti-tumor effects. Meanwhile, PGE2 inhibited mTOR–S6 signaling, causing impairment of the respiratory chain and accumulation of ROS, which triggered tumor-infiltrating lymphocyte ferroptosis.^41^ This study suggests that we can use COX inhibitors (*e.g.*, aspirin, ketorolac) or EP2/EP4 antagonists to block PGE2 signaling, which is highly expressed in prostate cancer. The decrease of PGE2 causes the decrease of GPX4 and the accumulation of ROS, which triggers ferroptosis in prostate cancer, and at the same time, it can restore the function of T cells, which can play a dual killing role.^41^^,^^42^

The tumor microenvironment profoundly influences ferroptosis sensitivity. Therapeutic strategies targeting the tumor microenvironment often aim to alleviate T cell dysfunction, amplify the immune–ferroptosis cycle, and reprogram immunosuppressive elements. Potential combinations include COX inhibitors with EP2/4 antagonists, anti-PD-1 with ACSL4 activators/GPX4 inhibitors, TauT inhibitors with CSF-1R inhibitors, and CXCR2 antagonists, among other multi-target synergistic approaches, to modify the immune environment and enhance tumor killing efficiency.

The interaction between intestinal flora and the immune microenvironment should not be ignored. Combined treatment with Icariin and curcumol deregulates immunosuppression and enhances CD8^+^ T-cell cytotoxicity by modulating the structure of the bacterial flora, inhibiting the metabolism of short-chain fatty acids, and down-regulating the epidermal growth factor receptor (EGFR)/signal transducer and activator of transcription 3 (STAT3)/programmed death-ligand 1 (PD-L1) axis.^43^

### Potential biomarkers of ferroptosis

As outlined in the preceding sections, the core pathways regulating ferroptosis and the key molecular mechanisms involved have been introduced. These represent potential targets for future research. Several targets, such as GPX4, have already been developed, with corresponding ferroptosis-promoting reagents now available, demonstrating significant promise. We have also compiled a list of promising biomarkers, including TFR1, ACSL4, SCD1, and Nrf2, summarizing their mechanisms of action across iron metabolism, lipid metabolism, and signaling pathways, aiming to provide insights for subsequent targeted drug development. Table 2 summarizes the characteristics, mechanisms, and functional evidence for these and other key biomarkers across iron metabolism, lipid metabolism, and signaling pathways.

**Table 2:** Potential biomarkers of ferroptosis.

Mechanism access	Specific factors	Mechanisms and values
Iron metabolism	TFR1/high level of iron	Risk of iron overload ↑ → sensitivity to ferroptosis inducers ↑
	NCOA4-FTH1 axial activity	
Lipid metabolism marker	ACSL4/PUFA	High expression of ACSL4/PUFA → lipid peroxidation susceptibility ↑
	SCD1/MUFA level	High expression of SCD1/MUFA level → lipid peroxidation susceptibility ↓
	PLA2G4A	PLA2G4A ↑ → PGE2 ↑ → anti-ferroptosis effect
	BH4 + CoQ10	BH4 + CoQ10 → anti-ferroptosis effect
Significant signaling pathways	PI3K–AKT–mTOR	PI3K–AKT–mTOR → inhibit ferroptosis
	Hippo/YAP	Bi-directional adjustment
	Nrf2	Nrf2 ↑ → enhances cellular antioxidant capacity
	PGE2	PGE2 ↓ → TIL ↑, GPX4 ↓
	SOX15	SOX15 ↑ → increase ferroptosis
Non-coding RNA	miR-9	miR-9↑ → GSH ↓
	miR-1252-5p	miR-1252-5p ↑ → FTH1 ↓
	LINC00336	LINC00336 ↑ → inhibit ferroptosis

### Targeted ferroptosis treatment strategies for prostate cancer

#### *Ferroptosis inducers and targeted ferroptosis drugs*

Ferroptosis inducers are small molecular compounds or biological factors that trigger ferroptosis. Ferroptosis inducers initiate cellular membrane damage and cell death by disrupting the cellular antioxidant defense system, promoting lipid peroxidation, and increasing intracellular iron content. Currently, ferroptosis inducers have shown great potential in prostate cancer research and have become a significant direction in anti-cancer drug development.

### Direct inhibition of GPX4 induces ferroptosis

GPX4 serves as a core regulatory factor in ferroptosis, maintaining cellular redox homeostasis by clearing lipid peroxides. It is a key target for targeting ferroptosis in prostate cancer treatment. RSL3, a classic inhibitor, irreversibly inhibits its antioxidant function by alkylating the selenium-containing cysteine at the active site of GPX4, resulting in lipid peroxide accumulation and triggering ferroptosis, *in vitro* experiments showed an inhibition rate of more than 50% in prostate cancer cells.^6^^,^^44^^,^^45^ In a subcutaneous xenograft model using TRAMP-C2 cells in C57BL/6 mice, RSL3 treatment significantly reduced tumor volume, and its combination with iron yielded the most potent anti-tumor efficacy.^46^ Its structural analogs ML162 and ML210 selectively inhibit GPX4 and also enhance the sensitivity of androgen receptor variants (AR-Vs)-mediated drug-resistant prostate cancer cells to ferroptosis.^47^ FIN56 exerts its effect through a dual mechanism: it promotes GPX4 degradation while activating the phospholipid biosynthesis pathway to increase the content of PUFAs in the cell membrane, which synergistically exacerbates lipid peroxidation damage. Additionally, altretamine (ALT) significantly enhances cancer cell sensitivity to ferroptosis by protein alkylation, destroying the GPX4 structure.^48^ These inhibitors target GPX4 through different molecular mechanisms, providing important directions for reversing prostate cancer resistance and developing combination therapies.

### Induction of ferroptosis via GSH depletion

GSH is a key cofactor for the antioxidant function of GPX4, and a decrease in its level can effectively induce ferroptosis in prostate cancer cells by indirectly inhibiting GPX4 activity. For example, Erastin inhibits cystine uptake by blocking cystine transporters (such as System Xc^−^), leading to reduced GSH synthesis, thereby inactivating GPX4 and triggering lipid peroxidation. Research also found that Erastin can interfere with prostate cancer cell growth, cell cycle, and DNA replication processes by regulating the expression of key genes and transcription factors.^4^, ^5^, ^6^ Experiments have shown that Erastin significantly reduced cell viability in DU145, PC3, 22Rv1, LNCaP, NCI–H660, ARCaP, C4-2, and LNCaP cell lines, demonstrating the broad spectrum of Erastin’s effects.^49^ Similar mechanisms are also observed in other compounds: the anti-inflammatory drug sulfasalazine (SAS) reduces cystine influx by competitively inhibiting SLC7A11, depleting GSH, and enhancing cancer cell sensitivity to chemoradiotherapy. The multi-kinase inhibitor sorafenib, while targeting the RAF/MEK pathway, also inhibits SLC7A11 expression, further lowering GSH levels to promote ferroptosis.^4^ Meanwhile, buthionine sulfoximine (BSO) directly blocks GSH biosynthesis by inhibiting γ-glutamylcysteine synthetase (GCL), weakening the antioxidant defense of cancer cells.^4^ These strategies all focus on the GSH–GPX4 axis, using differentiated pathways to synergistically enhance lipid peroxidation damage.^50^ Traditional Chinese medicine Aqueous-soluble sporoderm-removed *Ganoderma lucidum* spore powder (A-GSP) has also been found to inhibit GPX4 activity, leading to lipid peroxidation and ROS accumulation, showing promise for the development of traditional Chinese medicine in ferroptosis drugs.^51^

### Induction of ferroptosis by enhancing iron ion load

The abnormal accumulation of iron ions (Fe^2+^) promotes the generation of hydroxyl radicals (·OH) via the Fenton reaction, driving lipid peroxidation and exacerbating ferroptosis. Iron metabolism disorders in prostate cancer may increase cancer cells' sensitivity to this process. The androgen receptor (AR), a key regulatory factor in iron metabolism, may up-regulate iron storage proteins (such as ferritin) following androgen deprivation therapy, leading to iron ion accumulation in cancer cells, thus increasing susceptibility to ferroptosis. For example, PX-12 promotes iron ion uptake and increases ROS levels in cancer cells by inhibiting the thioredoxin system. For HepG2 cells, the IC50 was 30.30 μM at 24 h and 6.32 μM at 48 h, and ROS levels were significantly increased after PX-12 treatment compared to the control group.^52^ Additionally, iron-dependent drugs like dihydroartemisinin (DHA) and artemisinin (ART) form complexes with iron, further amplifying the Fenton reaction, increasing intracellular free iron concentrations, and exacerbating oxidative stress to induce castration-resistant prostate cancer cell death.^48^

### Induction of ferroptosis through other mechanisms

Several other ferroptosis inducers have shown multi-target regulatory potential in prostate cancer treatment. BAY 11–7085 up-regulates the key antioxidant stress enzyme HMOX1, promoting lipid peroxidation and directly driving ferroptosis. BAY 87–2243 enhances ferroptosis sensitivity by inhibiting mitochondrial complex I function and increasing ROS levels. Cil1 and Cil56 inhibit lipid peroxide generation and, in combination with α-tocopherol or deferoxamine (DFO), enhance antioxidant imbalance, making cancer cells more susceptible to ferroptosis. Conversely, MIR7 weakens ferroptosis by inhibiting the expression of lipid peroxidase-related enzyme arachidonate (12S)-lipoxygenase (ALOX12), a mechanism that may be related to prostate cancer radiotherapy resistance.^48^ Sanguinarine chloride down-regulates ubiquitin-specific peptidase 47 (USP47) and BTB and CNC homology 1 (BACH1) in a ROS-dependent manner, activating HMOX1 expression, and inducing ferroptosis through a dual pathway.^53^ Additionally, biflavonoids like robustaflavone have been shown to induce ferroptosis in breast cancer, and its analogs, ginkgetin and brachydins, though not directly verified in prostate cancer, may promote ferroptosis by ROS accumulation, inhibiting the PI3K–AKT–mTOR pathway, and activating the nuclear factor kappa B (NF-κB)-mediated tumor necrosis factor-alpha (TNF-α) signaling pathway.^54^ These studies reveal the potential therapeutic value of targeting lipid metabolism, oxidative stress, and inflammatory signaling to synergistically intervene in ferroptosis.

### Other targeted ferroptosis drugs

In addition to the above-mentioned ferroptosis-targeted drugs, numerous drugs targeting the ferroptosis process have been widely developed, such as various ferroptosis inducers, including Erastin, RSL3, and Artesunate. These agents act on targets such as SLC7A11, GPX4, FTH1, and Nrf2. They promote ferroptosis through multiple pathways, including inhibiting system Xc^−^, depleting GSH/CoQ10, blocking GPX4, or increasing free iron and ROS. A more comprehensive list of ferroptosis-inducing compounds, their targets, and mechanisms is provided in Table 3.

**Table 3:** Ferroptosis-inducing compounds and their mechanisms of action.

Compound	Target	Mechanism	References
Erastin	SLC7A11	Inhibits system Xc^−^, preventing cystine uptake and leading to glutathione (GSH) depletion	55,56
Sulfasalazine	SLC7A11	Inhibits system Xc^−^, preventing cystine uptake and leading to GSH depletion	57
Sorafenib	SLC7A11	Inhibits system Xc^−^, preventing cystine uptake and leading to GSH depletion	58
RSL3	GPX4	Covalently inhibits GPX4 activity, leading to lipid peroxide accumulation	59
FIN56	GPX4/CoQ10	Induces autophagy-mediated GPX4 degradation and depletes CoQ10	60
Artesunate	FTH1, NCOA4	Promotes degradation of FTH1 and NCOA4, elevating Fe^2+^ levels and decreasing GSH content	61
Dihydroartemisinin	GPX4, mTOR	Directly inhibits GPX4 and activates autophagy to degrade ferritin, elevating Fe^2+^ levels	62
Diallyl trisulfide	GPX4	Promotes ROS production and ferritin degradation, increasing the labile iron pool	63
Cisplatin	GSH	Induces GSH depletion, leading to lipid peroxidation	64
Curcumin	HO-1	Accumulates intracellular iron, increases ROS levels, and decreases GSH content	65
Flubendazole	p53	p53 binds to the SLC7A11 promoter region, inhibiting system Xc^−^ function	66
BSO	GSH	Inhibits glutathione synthesis, leading to GSH depletion	58
Glutamate	System Xc^-^	Competitively inhibits cystine uptake, reducing GSH levels	67
ML162	GPX4	Directly inhibits GPX4 activity	68
Brisatol/Trigonoline	Nrf2	Inhibits the Nrf2 pathway, increasing cellular iron levels	69
Lapatinib	Mitochondrial complex I	Inhibits mitochondrial complex I, leading to ROS accumulation and ferroptosis induction	70

### Combination therapy strategies

Ferroptosis inducers can be used in combination with anti-androgen drugs such as enzalutamide and bicalutamide.^17^^,^^38^ These anti-androgen drugs inhibit AR signaling, reducing GPX4 expression and weakening the cell’s antioxidant capacity. For example, the combination of enzalutamide or RSL3 with anti-androgen drugs significantly inhibits the growth and migration of CRPC cells.^46^ New AR inhibitors, such as darolutamide, combined with ferroptosis inducers, can also induce ferroptosis by inhibiting the SREBP1–FASN axis and increasing PUFA content.^71^

Ferroptosis inducers can also be used in combination with chemotherapy drugs. RSL3, when used in combination with cisplatin, synergistically inhibits the viability and proliferation of prostate cancer cells at lower doses in both *in vitro* and *in vivo* models. The ferroptosis inducer can reverse the drug resistance caused by the binding of cisplatin to GSH and reduce toxicity.^4^^,^^7^ Docetaxel, which induces apoptosis by stabilizing microtubules, down-regulates GPX4 and SLC7A11, synergistically enhancing the action of inducers.^7^^,^^72^ In docetaxel-resistant PC3 cells, Erastin and RSL3 can reverse docetaxel resistance, significantly inhibit cell proliferation, and the combination of RSL3 and docetaxel significantly inhibits tumor growth, and Erastin can significantly lower the IC50 value of cells to docetaxel, from 355.97 ± 66.89 nM to 32.94 ± 6.01 nM in ovarian cancer, suggesting that it may have the same effect in prostate cancer.^72^^,^^73^ In addition to chemotherapy, combining radiation therapy with ferroptosis has also been shown to significantly inhibit prostate cancer growth.^17^

Ferroptosis inducers combined with targeted metabolic drugs can lead to tumor regression. mTORC1 inhibitors (such as CCl-779) combined with RSL3 have been shown to have better anti-cancer effects in PTEN-deficient prostate cancer.^23^ CCl-779 inhibits mTORC1, reduces SREBP1 nuclear translocation, decreases FASN expression, increases the proportion of PUFAs, and, when used in combination with RSL3, enhances lipid oxidation and promotes ferroptosis. Experimental results demonstrate that the combined use of CCl-779 and RSL3 significantly reduces tumor cell survival rates, with a reduction of nearly 50%.^74^ PHGDH inhibitors (such as NCT-503) combined with enzalutamide can enhance CRPC cell sensitivity to ferroptosis.^38^ The core idea behind these strategies is to promote ferroptosis by directly acting on the final pathway of lipid peroxidation through two or more ferroptosis inducers. This combination approach effectively overcomes the problem of single-drug resistance and significantly improves treatment efficacy.

Immunotherapy strategies can treat prostate cancer by improving the tumor’s immune microenvironment. PD-1 is a transmembrane protein mainly expressed on activated T cells, B cells, NK cells, and other immune cells. Anti-PD-1/PD-L1 antibodies relieve T-cell suppression, and CD8^+^ T cells secrete IFN-γ to inhibit SLC7A11, enhancing ferroptosis sensitivity.^17^^,^^39^ DECR1, a direct target gene of AR, may enhance prostate cancer proliferation. The use of DECR1 inhibitors combined with immunotherapy has become one of the new therapeutic approaches.^19^

Iron overload can also be a combination therapeutic strategy. For instance, iron-dextran and ferric carboxymaltose can induce ferroptosis in prostate cancer cells and enhance the efficacy of the anti-androgen drug bicalutamide.^17^ Additionally, various other combination therapies have achieved certain therapeutic effects, such as mitochondrial electron transport chain ETC inhibitors combined with ACSL4 gene therapy, AR inhibitor NEO2734 combined with enzalutamide.^17^ Ceapin-A7, an ATF6α inhibitor, modulates PLA2G4A and enhances the efficacy of enzalutamide combination therapy.^30^^,^^75^ These therapies provide new approaches for prostate cancer treatment.

### Nanomedicine and targeted therapy

Drug targeting is also crucial in prostate cancer treatment, as high targeting efficiency can yield twice the result with half the effort. In the treatment of prostate cancer, especially CRPC, nanotechnology has shown potential by enabling precise drug delivery and synergistic drug mechanisms. MPDA/Fe/RSL3@M-gy1 nanoparticles, using mesoporous polydopamine (MPDA) as a carrier, load Fe^3+^ and ferroptosis inducer RSL3 through π-π stacking and hydrophobic interactions. The surface-modified prostate-specific membrane antigen (PSMA) targeting antibody fragment (gy1) specifically recognizes CRPC cells for targeted delivery. These nanoparticles release Fe^3+^ and RSL3 in the acidic environment of the lysosome, generating ROS through the Fenton reaction of Fe^3+^ and inhibiting GPX4 activity, significantly inducing lipid peroxidation and ferroptosis, thereby inhibiting CRPC tumor growth and bone metastasis in *in vivo* experiments, with no systemic toxicity observed. MPDA/Fe/RSL3@M-gy1 nanoparticles also have extremely low IC50 values and exhibit superior properties.^76^ Another magnetic lipid nanoparticle (t-ML) utilizes an external magnetic field for targeted delivery, co-loading high-γ-linolenic acid (DGLA) and DECR1 siRNA. DGLA promotes lipid peroxidation by increasing PUFA content, while DECR1 siRNA knocks down PUFA degradation enzyme DECR1 to suppress metabolic feedback. Together, they synergistically enhance ferroptosis sensitivity.^21^ Additionally, liposome-coated manganese sulfide nanoparticles (Lpo@MnS-GOx) surface-conjugated with glucose oxidase (GOx) catalyze the generation of hydrogen peroxide (H_2_O_2_) in the tumor microenvironment, driving the Fenton-like reaction of Mn^2+^ to generate hydroxyl radicals (·OH). At the same time, manganese sulfide releases hydrogen sulfide (H_2_S), which, in combination with H_2_O_2_, intensifies ROS accumulation, thus inducing ferroptosis and inhibiting cancer cell proliferation.^9^

p-PSMA-CAR-NK92MI cell therapy is a therapy that targets NK cells. p-PSMA-CAR-NK92MI cells can selectively kill PSMA-positive target cells, and after co-cultivation with PSMA-positive target cells, they can significantly increase the concentration of IFN-γ, TNF-α, and granzyme B, and significantly inhibit the growth of tumors.^77^

These emerging nanomedicine strategies provide high-efficiency and low-toxicity innovative directions for prostate cancer treatment through targeted delivery and drug synergy.

### Advantages of novel therapies for the treatment of castration-resistant prostate cancer

The main pathological type of prostate cancer is vesicular cancer, with other subtypes being relatively rare. Currently, the main treatments for this disease are still limited to radical surgery and radiation therapy, while drug development for emerging targets is lagging. Lowering androgen levels in patients is a common strategy to slow the progression of prostate cancer. However, the vast majority of patients who undergo androgen deprivation therapy continue to progress even after serum testosterone has been reduced to nadir levels (commonly defined as < 50 ng/dL), a stage defined as CRPC.

Given the limited efficacy of conventional therapies for CRPC, emerging targeted therapeutic strategies have shown significant benefits. For example, a regimen combining iron supplementation, RSL3 (a ferroptosis inducer), and enzalutamide (an androgen receptor antagonist) resulted in a significant improvement in pathology: the percentage of cancerous areas was reduced from 47.5% to 16.4%, while the percentage of normal prostate tissue increased from 28% to 62%.^46^ In addition, targeted knockdown of the key gene DECR1 resulted in a 40% reduction in primary tumor volume and an 80% reduction in lung metastases in the experimental model.^19^ These novel therapies developed based on the ferroptosis mechanism offer the potential to prolong the survival of patients with advanced CRPC.

### Challenges and the future

#### *Drug resistance*

In the therapeutic landscape of prostate cancer, ferroptosis, an emerging form of regulated cell death, presents a promising avenue for disease management. However, as research progresses, the issue of drug resistance in ferroptosis-based therapies has become increasingly prominent, posing a critical barrier to its clinical application.

Prostate cancer cells can up-regulate the expression of ferroptosis-suppressing proteins through multiple mechanisms. For instance, FSP1 can reduce CoQ10 on the cell membrane to ubiquinol (CoQH2), thereby capturing free radicals and protecting cells from ferroptosis.^78^ Overexpression of Nrf2 can also up-regulate the expression of antioxidant enzymes and inhibit ferroptosis. Abnormal activation of specific signaling pathways in prostate cancer cells leads to the overexpression of GPX4, allowing cancer cells to resist ferroptosis-inducing therapies.^79^ Additionally, solute carrier family 7 member 11 (SLC7A11) is frequently overexpressed, further enhancing cancer cell resistance to ferroptosis.^17^^,^^72^ In addition, a transsulfuration pathway exists that can bypass the Xc^−^ system to synthesize cysteine, thereby weakening the efficacy of SLC7A11 inhibitors.^80^^,^^81^ Compensatory or disrupted key regulatory relief of lipid metabolism can interrupt the progression of ferroptosis, leading to drug resistance. The unique vulnerability of prostate cancer has given rise to multiple drug resistance pathways.

Prostate cancer cells exhibit aberrant activation of specific pathways that suppress ferroptosis. For instance, in the AKT–S-phase kinase-associated protein 2 (SKP2)–ACSL4 pathway, excessive phosphorylation of AKT promotes the degradation of ACSL4. This impairs the activation of PUFAs into their corresponding acyl-CoA derivatives, thereby reducing the synthesis of PUFA-containing phosphatidylethanolamines (PUFA-PEs), which are critical for ferroptosis execution.^82^ In the mouse double minute 2 (MDM2)–tropomyosin receptor kinase A (TrkA)–alkylglycerone phosphate synthase (AGPS) axis, AGPS regulates ferroptosis by modulating peroxisomal function. However, TrkA, through phosphorylation of AGPS, facilitates its binding to MDM2, inhibiting AGPS function. This disrupts peroxisomal function, exacerbates lipid metabolic dysregulation, destabilizes redox balance, and inhibits ferroptosis induction, ultimately leading to drug resistance.^47^

Furthermore, the tumor microenvironment plays a critical role in ferroptosis resistance. The hypoxic tumor microenvironment of prostate cancer triggers adaptive responses that promote resistance. Hypoxic conditions induce increased expression of hypoxia-inducible factors (HIF). The stable expression of HIFs activates genes involved in iron metabolism, oxidative stress responses, and antioxidant defense systems,^37^ allowing cancer cells to maintain redox homeostasis and evade ferroptosis under therapeutic stress. Furthermore, immune cells such as tumor-associated macrophages infiltrating the tumor microenvironment secrete cytokines and growth factors,^37^ which activate pro-survival signaling pathways in cancer cells, indirectly fostering ferroptosis-resistant phenotypes.

Adaptive responses and compensatory mechanisms are important mechanisms underlying ferroptosis resistance in prostate cancer. Future research should further elucidate additional resistance mechanisms and explore new combination therapy strategies to overcome emerging resistance issues and enhance the efficacy of ferroptosis therapy in prostate cancer.

### Issues concerning targeting precision

In the field of ferroptosis therapy for prostate cancer, targeted precision has also become a core element determining its therapeutic effect and clinical application prospects. The regulatory mechanisms of ferroptosis involve a large number of complex and interrelated molecular pathways. How to precisely target key nodes is a major challenge and a hot research topic at present.

Firstly, the heterogeneity of prostate cancer is a major obstacle to achieving precise targeted ferroptosis therapy. There are significant differences in the expression and functional status of ferroptosis-related molecules among prostate cancer cells from different patients, and even among different cell subpopulations within the same tumor. For example, some prostate cancer cells may rely on the high expression of SLC7A11 to maintain intracellular GSH levels, thereby resisting ferroptosis, while other cells may inhibit lipid peroxidation by up-regulating the activity of GPX4.^72^ This heterogeneity requires that when designing ferroptosis-targeted therapeutic strategies, the individual differences of patients and the diversity of tumor cells must be fully considered to achieve precise classification and personalized treatment.

Secondly, the functions of ferroptosis-related molecules in the occurrence and development of prostate cancer are complex. Taking iron metabolism-related proteins as an example, they are not only involved in the regulation of ferroptosis, but also play roles in multiple biological processes such as the proliferation and migration of tumor cells. This means that simply targeting a certain ferroptosis-related molecule may trigger a series of unexpected biological effects, affecting the precision and safety of treatment. For instance, excessive inhibition of iron uptake proteins may promote ferroptosis, but it may also interfere with the iron homeostasis of normal cells, causing adverse reactions such as anemia.

In addition, existing models are unable to adequately simulate the human tumor microenvironment. Current understanding of ferroptosis primarily relies on studies using traditional cell lines and animal models, neither of which can accurately reflect the complexity of the tumor environment in human prostate cancer. To overcome this issue, preclinical systems that better mimic, such as patient-derived organoids and xenografts, need to be introduced in the future. It is worth noting that organoid Models provide a complex platform that more accurately reflects iron death therapy in clinical settings, but at the same time, complex pathways and phenomena will be observed.^83^

Lastly, there is currently a lack of integration of multi-omics data to identify precise targets. Only with more precise targets can treatment outcomes be optimized and side effects minimized. Integrating multi-omics technologies, including transcriptomics, lipidomics, redox proteomics, and single-cell sequencing, is crucial for comprehensively analyzing the actionable molecular features within the AR-ferroptosis regulatory network. These methods not only aid in identifying new biomarkers predictive of treatment response but may also reveal more complex interactions between ferroptosis susceptibility and AR signaling dynamics.

### Other issues facing clinical translation

Despite the potential value of ferroptosis in the treatment of prostate cancer, especially in CRPC and drug-resistant subtypes (such as androgen deprivation therapy-resistant prostate cancer), its clinical translation still faces multiple challenges. At the level of mechanism research, the GPX4-independent regulatory pathways have not been fully elucidated. For example, membrane-bound glycerophospholipid O-acyltransferase 1/2 (MBOAT1/2) can regulate ferroptosis independently of GPX4 through phospholipid remodeling, but its role in prostate cancer still needs to be verified.^75^ Meanwhile, the interaction mechanism between hormone signaling and ferroptosis is still controversial. For instance, the androgen receptor may inhibit ferroptosis by up-regulating MBOAT2, while anti-androgen drugs (such as enzalutamide) may enhance ferroptosis sensitivity by down-regulating MBOAT2. Preclinical studies are mostly limited to cell and mouse models. For example, the GPX4 inhibitor JKE-1674 has shown tumor-inhibiting effects in prostate cancer mouse models with retinoblastoma transcriptional corepressor 1 (RB1) deficiency, but its clinical safety and indications still need systematic evaluation.^84^ The heterogeneity of biomarker screening further increases the complexity. The expression of core regulatory factors such as GPX4, SLC7A11, and ACSL4 varies significantly among different prostate cancer subtypes. For example, high expression of ACSL4 in RB1-deficient prostate cancer can enhance susceptibility to ferroptosis, while the roles of GPX4-dependent and GPX4-independent defense mechanisms (such as the FSP1, DHODH, and GCH1 pathways) have not reached a consensus.^17^

In addition, the reliability of existing biomarkers in predicting treatment response is insufficient. For example, the UBI inhibitor leflunomide has shown activity in PTEN-mutated prostate cancer cells, but its clinical application effect still needs to be verified. In terms of precision medicine integration, there is a lack of clear criteria for patient selection. For example, although high expression of FSP1 is associated with poor prognosis of prostate cancer, individualized strategies based on this have not been established.^17^ Combined treatment strategies also need to be optimized. Although the synergistic potential of ferroptosis and immune checkpoint inhibitors (such as PD-1/PD-L1 antibodies) has been proposed, relevant clinical data are still extremely lacking.^39^ These limitations highlight the systematic difficulties of ferroptosis from cutting-edge mechanism exploration to clinical practice implementation.

Unfortunately, current experiments related to ferroptosis remain confined to cell lines and animal models, failing to advance to the clinical stage. Moreover, innovative experiments have scarcely been conducted. This necessitates future researchers to undertake more relevant studies and expedite the clinical application of ferroptosis agents. We have compiled existing relevant experiments in Table 4 for reference, aiming to inspire future researchers.

**Table 4:** Ongoing clinical trials.

ClinicalTrials.gov ID	Experimental phase	Arms and interventions	Primary outcome measures	Secondary outcome measures	Preliminary results/expected timeline
NCT03834493	Phase Ⅲ	Pembrolizumab plus enzalutamide	Overall survival, radiographic progression-free survival (PFS) per prostate cancer Working group (PCWG)-Modified response evaluation criteria in solid tumors Version 1.1 (RECIST 1.1) as assessed by Blinded independent central review	Time to initiation of the first subsequent anti-cancer therapy or death, prostate-specific antigen (PSA) response rate, PSA undetectable rate	Scheduled completion: May 29, 2026
NCT04951492	Phase Ⅱ	Olaparib	Lowest on-treatment PSA	Overall response rate, radiographic PFS, PSA, PFS, overall survival	Patients achieving at least a 50% decline in PSA received at least 12 weeks of olaparib.
NCT02905318	Phase Ⅱ	Palbociclib	Clinical benefit rate estimated by the proportion of evaluable patients who had complete response, partial response, or stable disease as their best response to treatment	Objective response determined by RECIST 1.1, PFS, and overall survival	Expected completion: December 30, 2025
NCT00090545	Phase Ⅱ	BAY 43-9006	PFS	Number of participants with adverse events, median overall survival, and overall response evaluated by RECIST	No results posted.
NCT00430235	Phase Ⅱ	BAY 43–9006 (400 mg, orally, twice daily in a 28-day cycle (morning and evening)), bicalutamide (50 mg, orally, once daily in a 28-day cycle (morning))	Rate of PSA-response	Time to treatment failure, time to PSA progression, duration of PSA response	Sorafenib combined with bicalutamide demonstrates a certain PSA response rate in chemotherapy-naive patients with hormone-refractory prostate cancer.
NCT02204072	Phase Ⅰ	BI 836845 plus enzalutamide	Phase Ib escalation part: number of patients with dose-limiting toxicities; phase Ib escalation part: maximum tolerated dose based on the occurrence of dose-limiting toxicities during the first treatment course; phase Ib expansion part: PSA response; phase II part: PFS based on investigator assessment	Phase Ib expansion part: PFS based on investigator assessment; phase Ib expansion part: changes in circulating tumor cell response (circulating tumor cell reduction from ≥ 5 to < 5 cells per 7.5 mL blood for at least one post-baseline time point); phase II part: radiological PFS based on central review	BI836845 plus enzalutamide demonstrated acceptable safety in metastatic castration-resistant prostate cancer (mCRPC) and showed dual PSA and imaging response activity, with a particularly pronounced trend toward benefit in the IGF-high population.
NCT01215032	Phase Ⅱ	Metformin	PSA response	Number of participants with PSA response, relationship between baseline metabolomic profile and PSA response, and percent maintaining glycemic control	Metformin monotherapy demonstrated limited anti-tumor activity (PSA50 only 11%) in chemotherapy-naive mCRPC patients, with no imaging-based objective responses observed. Therefore, it is not recommended as first-line monotherapy.
NCT05488548	Phase Ⅰ	NEO2734	Maximum tolerated dose, dose-limiting toxicities, recommended phase 2 dose		Phase I expanded cohort (mCRPC, *n* ≈ 40) is currently recruiting, with preliminary overall response rate and radiographic PFS data expected in Q4 2025.
NCT03275857	Phase Ⅰ	Cisplatin	Response to dosing differences of cisplatin from lab and scan results; toxicity observed with dosing differences of cisplatin		No results posted.

### Clinical translation of ferroptosis in prostate cancer

#### *Development of mechanism-driven biomarkers*

There are numerous biomarkers involved in prostate ferroptosis, with lipid peroxidation being a key step. ACSL4 catalyzes the formation of PUFA-CoA, promoting the integration of PUFAs into membrane phospholipids, making it an important biomarker. Therefore, we can innovatively use the “ACSL4 immunohistochemical scoring” method to monitor and predict the response and efficacy of ferroptosis inhibitors. Another example is the inactivation of the Hippo pathway, which leads to the accumulation of downstream YAP. We can develop a “YAP nuclear localization index” as a screening criterion for treatment protocols to determine whether patients are suitable for corresponding ferroptosis treatment methods. Furthermore, tracers can be designed based on the key ferroptosis molecule PUFA, such as ^18^F-labeled PUFA analogues, to monitor lipid peroxidation levels in tumors in real time, providing a visual model for treatment assessment. Additionally, direct lipidomic analysis of prostate cancer cells can be performed to measure their PUFA content. This approach may prove more cost-effective and efficient. Finally, we can develop antibody-drug conjugates targeting specific receptors such as SLC7A11. These conjugates precisely identify antigens on the surface of cancer cells and combine with potent drugs to strike and eliminate prostate cancer cells. To deliver precise treatment to patients and target cancer with accuracy, while avoiding severe side effects caused by tumor heterogeneity, the development of biomarkers provides rigorous and precise criteria for patient stratification. Researchers have stratified patients based on negative regulators of ferroptosis, as shown in Table 5, which may offer valuable reference for future patient stratification.^85^

**Table 5:** Biomarker-driven patient stratification.

Biomarkers	Level of expression	Molecular function	Clinical significance	Treatment strategy
SLC7A11	High expression	Cystine transporter; maintaining GSH synthesis	Induce docetaxel resistance; promote tumor metastasis and castration-resistant prostate cancer progression	Combined with SLC7A11 inhibitors (Erastin/IKE/sorafenib) or radiotherapy sensitization
GPX4	High expression	Reducing lipid peroxides and inhibiting ferroptosis	Enhance radiotherapy resistance; protect cancer cells from oxidative damage	GPX4 inhibitors (RSL3/ML162/Withaferin A) combined with radiotherapy
DECR1	High expression	Rate-limiting enzyme that promotes PUFA oxidation	Shorten disease-free survival; inhibit ferroptosis and promote castration-resistant prostate cancer cell proliferation	DECR1 siRNA combined with ferroptosis inducers (*e.g.*, FIN56)
PANX2	High expression	Channel protein; up-regulates SLC7A11 expression	Correlate positively with Gleason score; inhibit ferroptosis via the NRF2/STAT3 pathway	PANX2 silencing therapy combined with antioxidant therapy
HSPB1	High expression	Heat shock protein; blocks iron uptake and lipid ROS generation	Inhibit Erastin-induced ferroptosis, associated with invasive cancer and poor prognosis	HSPB1 inhibitors combined with ferroptosis inducers
PI3K/AKT/mTOR	Activation	Signaling pathway; inhibits lipid peroxidation via SCD1/NRF2	Promote castration-resistant prostate cancer progression and castration resistance; inhibit ferroptosis	mTOR inhibitors (*e.g.*, everolimus) combined with ferroptosis inducers

### Optimization of combination strategies guided by drug resistance mechanisms

Ferroptosis therapy requires addressing drug resistance issues to achieve clinical translation. Designing combination strategies based on molecular mechanisms is the key to overcoming drug resistance. For the drug resistance mechanism involving FSP1-CoQ10 up-regulation, FSP1 inhibitors can be combined with GPX4 inhibitors (such as RSL3), and a liposomal co-delivery system can be used to achieve simultaneous release within tumors, thereby overcoming single-agent resistance.^78^ To address resistance issues related to the transsulfuration pathway, combining CBS inhibitors (PEG-modified asparaginase) with Xc^−^ inhibitors (Erastin) can inhibit cystathionine synthesis, thereby blocking GSH synthesis. The cyclooxygenase 2 (COX-2)–PGE2 axis mediates dual resistance by inhibiting T cell function and promoting GPX4 expression. An innovative triple therapy combining EP4 antagonists (Grapsiprant) with PD-1 antibodies and ferroptosis inducers can be employed to eliminate immune suppression while triggering tumor-specific lipid peroxidation.^41^ For each resistance mechanism, corresponding targeted strategies should be sought to address it. The rational combination and use of these strategies can also reduce the IC50 of various drugs, minimize toxicity risks for patients, maximize the killing of prostate cancer cells, and increase the likelihood of survival.

### Nanotechnology advances clinical translation

PSMA-targeted nanoparticles (such as MPDA/Fe/RSL3@M-gy1) concentrate drugs at tumor sites, reducing systemic toxicity. Their development has been completed, but their safety in non-human primates remains to be investigated. Secondly, their penetration efficiency in solid tumors may be insufficient. Additionally, PSMA expression in prostate cancer may exhibit spatial heterogeneity, with some subpopulations showing low PSMA expression, potentially leading to heterogeneous escape from the target. To overcome these issues, variable-sized nanoparticles can be developed to increase permeability, improve drug utilization, and develop new targets on the membrane. The redeveloped nano-targeted particles are expected to simultaneously perform the “recognition-killing-immune activation” cycle. The advancement of nano-precision technology can break through clinical bottlenecks and open up a new path of “precision delivery, toxicity shielding, and integrated diagnosis and treatment”.

### Possible directions for future research

The mechanism of ferroptosis has shown great promise in prostate cancer treatment, and future research should focus on the following interrelated directions: first, it is necessary to deeply analyze the resistance mechanism to existing ferroptosis inducers, such as Erastin, and to identify key molecular targets, such as exploring the role of knockdown of ferritin heavy chain 1 pseudogene 8 (FTH1P8) in the reversal of resistance to docetaxel, to lay the foundation for overcoming drug resistance and prolonging the efficacy of the drug.^16^ Second, it is crucial to explore strategies to enhance the efficacy of ferroptosis induction, including the development of targeted delivery systems to achieve precise drug localization, the optimization of drug molecular structure to screen for highly sensitive inducers, and the design of spatiotemporally controlled therapies to enhance the intensity and durability of local effects.^21^ Third, for the new ferroptosis-related targets that have been discovered but not yet fully exploited, the three-dimensional crystal structure analysis should be accelerated to guide the rational design of highly specific targeted drugs.^19^ Fourth, the clinical value of key molecules of the ferroptosis pathway as prognostic biomarkers should be systematically evaluated, for example, the association between high expression of Holliday junction recognition protein (HJURP) or peroxiredoxin-1 (PRDX1) as well as cyclin-dependent kinase 12 (CDK12) deletion and patients' poor survival prognosis should be investigated, and prognostic prediction models based on the ferroptosis pathway should be constructed in an integrated manner to more accurately assess the survival outcomes of patients.^84^^,^^86^ Fifth, robust predictive biomarkers can be developed for identification and validation, and omics data can be analyzed and integrated using machine learning models to predict biomarkers sensitive to ferroptosis from biopsy samples, thereby constructing updated and improved scoring prediction models. Sixth, rational combination regimens based on mechanism-driven synergy can be developed, grounded in a robust understanding of mechanism-driven synergy, such as deeply inhibiting AR signaling or synergistically enhancing immunotherapy. Seventh, advanced imaging technologies can be developed to monitor ferroptosis in real-time, such as lipid peroxidation molecular probes and chemical exchange saturation transfer imaging (magnetic resonance imaging), to non-invasively detect the oxidation state of PUFAs in tumor regions, providing imaging evidence for combination therapy regimens. Finally, there is an urgent need to deeply investigate the remodeling effects of ferroptosis activation, such as targeted knockdown of DECR1 on the tumor immune microenvironment, and to evaluate the synergistic anti-tumor potential of corresponding ferroptosis inducers, such as DECR1 inhibitors, in combination with immunotherapy to provide more effective treatment strategies for advanced patients.

Ferroptosis has opened up a new pathway for prostate cancer treatment by targeting GPX4, iron metabolism, and the immune microenvironment, effectively overcoming traditional treatment resistance. However, the complexity of drug resistance mechanisms, the lack of targeting precision, and the difficulty of clinical translation are still the main challenges. In the future, we need to explore the non-GPX4 pathway, develop precise biomarkers and optimize the combination strategy, and break through the bottleneck with the help of nanotechnology and gene editing. With the mechanism analysis and technological innovation, ferroptosis is expected to move from the laboratory to the clinic, providing a new hope for the eradication of prostate cancer.

## CRediT authorship contribution statement

**Yixiang Huang:** Writing – review & editing, Writing – original draft, Conceptualization. **Yuanxin Ma:** Writing – review & editing, Writing – original draft, Conceptualization. **Jiachen He:** Writing – review & editing, Writing – original draft, Conceptualization. **Tanjing Song:** Writing – review & editing, Conceptualization.

## Conflict of interests

The authors declare that there is no conflict of interests.
